# A Liquid Chromatographic Method for Rapid and Sensitive Analysis of Aflatoxins in Laboratory Fungal Cultures

**DOI:** 10.3390/toxins12020093

**Published:** 2020-01-30

**Authors:** Ahmad F. Alshannaq, Jae-Hyuk Yu

**Affiliations:** Department of Bacteriology, University of Wisconsin-Madison, 1550 Linden Drive, Madison, WI 53706, USA

**Keywords:** aflatoxins, laboratory culture, extraction, HPLC, recovery, detection limits

## Abstract

Culture methods supplemented with high-performance liquid chromatography (HPLC) technique provide a rapid and simple tool for detecting levels of aflatoxins (AFs) produced by fungi. This study presents a robust method for simultaneous quantification of aflatoxin (AF) B1, B2, G1, and G2 levels in several fungal cultivation states: submerged shake culture, liquid slant culture, and solid-state culture. The recovery of the method was evaluated by spiking a mixture of AFs at several concentrations to the test medium. The applicability of the method was evaluated by using aflatoxigenic and non-aflatoxigenic *Aspergilli*. A HPLC coupled with the diode array (DAD) and fluorescence (FLD) detectors was used to determine the presence and amounts of AFs. Both detectors showed high sensitivity in detecting spiked AFs or AFs produced in situ by toxigenic fungi. Our methods showed 76%–88% recovery from medium spiked with 2.5, 10, 50, 100, and 500 ng/mL AFs. The limit of quantification (LOQ) for AFs were 2.5 to 5.0 ng/mL with DAD and 0.025 to 2.5 ng/mL with FLD. In this work, we described in detail a protocol, which can be considered the foremost and only verified method, to extract, detect, and quantify AFs employing both aflatoxigenic and non-toxigenic *Aspergilli*.

## 1. Introduction

Aflatoxins (AFs) are a group of mycotoxins that are toxic, carcinogenic, and mutagenic. Amongst them, aflatoxin B1 (AFB1) is the most potent carcinogen found in nature and thus is classified as a group 1 carcinogen to humans by the International Agency for Research on Cancer (IARC) [1,2]. AFs are produced mainly by a common fungus *Aspergillus flavus* in fields, transportation, and storage conditions. AFs consistently and increasingly contaminate both human food and animal feed, and thus have been strictly regulated by the government authorities in over 100 countries in the world [3,4,5]. Trace levels of AFs, 4–20 parts per billion (ppb), can be considered hazardous, and foods with higher amounts are not fit for human consumption [6]. As global warming progresses, AF-producing molds will expand their growing regions, leading to an increased burden of AF contamination in the world [7,8,9].

**Figure 1:**
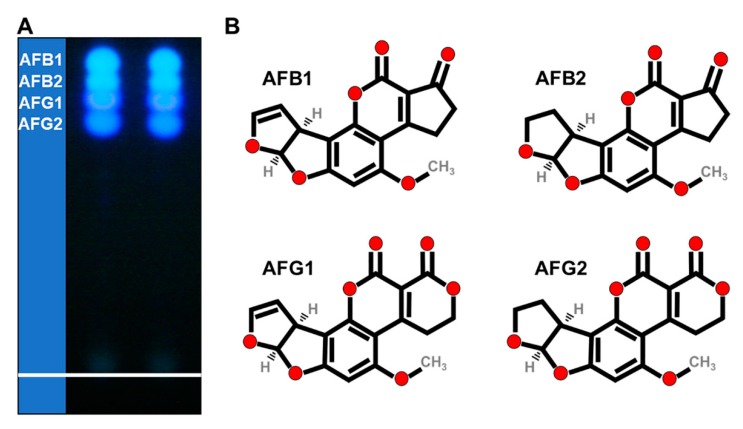
Aflatoxin (AF) thin layer chromatography (TLC) and structures. (**A**) A thin layer chromatograph of standard aflatoxin mixture containing AFB1, AFB2, AFG1, and AFG2. Note the color and the separation order. The photo was taken in a UV chamber at 365 nm. (**B**) Chemical structure of AFB1, AFB2, AFG1, and AFG2.

AFs are fluorescent heterocyclic secondary metabolites with molecular weights of 286 to 346 Da. Although more than 13 types of AFs have been discovered, AFB1, AFB2, AFG1, AFG2, and AFM1 (in milk) are particularly hazardous to humans and animals, as they have been commonly present in food and feed. The “B” and “G” refer to the blue and green-blue fluorescent colors emitted under ultraviolet (UV) light (Figure 1A), and the numbers represent the travelled position from the front line on the thin layer chromatography (TLC); moreover, AFB2 and AFG2 are the dihydroxy derivatives of AFB1 and AFG1, respectively [10,11]. Due to their oxygenated pentaheterocyclic structure, which is known as coumarinic nucleus, AFs have natural fluorescence properties (Figure 1A,B). This ability to fluoresce has paved the way for most analytical methods for the detection and quantification of these toxins [12]. Because of the absence of a double bond in the furan ring, AFB2 and AFG2 have a higher fluorescence quantum yield of fluorophore than the unsaturated compounds AFB1 and AFG1 [13].

*Aspergilli* residing in field soil of *A. flavus* specifically, is considered as the main source of AF contamination of agricultural products; however, not all strains of *A. flavus* produce AFs [14]. Communities of AF-producing fungal residents in varying agricultural environments are complex groups of diverse individuals. Thus, knowing the AF-producing potential of *A. flavus* populations is an important factor for the predicting the incidence and severity of AF contamination. On the other hand, although it was thought that *A. flavus* only produced B type AFs, recent reports have demonstrated that several *A. flavus* strains can also produce the G type AFs [15,16,17,18].

To detect and differentiate aflatoxigenic and non-toxigenic *Aspergilli*, several methods have been developed including molecular marker-based methods and fungal culture methods [19,20]. Currently, in most cases, aflatoxigenic fungi are being identified by culture methods coupled with thin layer chromatography (TLC) or high-performance liquid chromatography (HPLC). However, to the best of our knowledge, no methods have been optimized and validated for simultaneous quantifications of aflatoxin cocktail (AFB1, AFB2, AFG1, AFG2) in the fungal cultures. Gell and Carbone have used HPLC-FLD (fluorescence) for quantification of AFB1 from fungal mycelium culture after sample purification by solid phase extraction tubes (SPE), and they were able to achieve a limit of detection (LOD) and limit of quantification (LOQ) of 2 and 3.9 ng/mL, respectively [21]. Culture method has a number of advantages over others, including it being inexpensive, rapid, available in most labs, and requiring minimal technical skills. However, due to the lack of verifications of these methods, they are generally regarded as being less precise than the other methods. Here, we report a new method for a rapid, sensitive, and simultaneous detection of AFB1, AFB2, AFG1, and AFG2 produced in laboratory culture conditions using HPLC equipped with a conventional diode array (DAD) and a fluorescence detector (FLD) without using any pre- or post-column derivatization reagents, SPE and immune affinity column (IAC), or fluorescent enhancers. Moreover, we have optimized and validated the method through a series of experiments to meet the research laboratory needs for a robust, fast, easy to use, cheap, and environmentally friendly protocol with minimum organic solvents waste.

## 2. Results and Discussion

Sample preparation plays a key role for the quality of chromatographic results. The selection of extraction solvent and condition are very important for achieving the true value of the assigned analyte. Prior to validation of the method, we optimized the AF extraction efficiency conditions by testing the effect of different extraction solvents, the effect of extraction solvent amount, and the effect of shaking time (unpublished data). We tested five solvents: chloroform, ethyl acetate, acetone, petroleum ether, and methanol in six different sample to solvent ratios (1:1, 1:1.5, 1:2, 1:2.5, 1:3, and 1:5). We also assessed the effect of the shaking time by vortexing the samples for 30, 60, 90, 120, 150, and 300 s. Through it all, we found that chloroform and ethyl acetate were the best extraction solvents with the highest recovery values. The data revealed that the extraction yield with chloroform was a little higher, with no significant differences, when compared with ethyl acetate. We chose chloroform as the AF extraction solvent in this study because (1) higher recover values were achieved and (2) AFs are more stable and soluble in chloroform than in ethyl acetate [22]. Technically, complete obtaining of the lower organic layer (chloroform) was achievable and easier than those on the top (ethyl acetate). We also found that a minimum 1.5-fold volume of chloroform and 30 s shaking time produced the maximum AF recovery values. Results indicated that total transfer of AFs can be accomplished by two extractions with chloroform. Although there was no detectable AFs in the third chloroform extract, three extractions are recommended to preclude loss of toxin.

**Figure 2:**
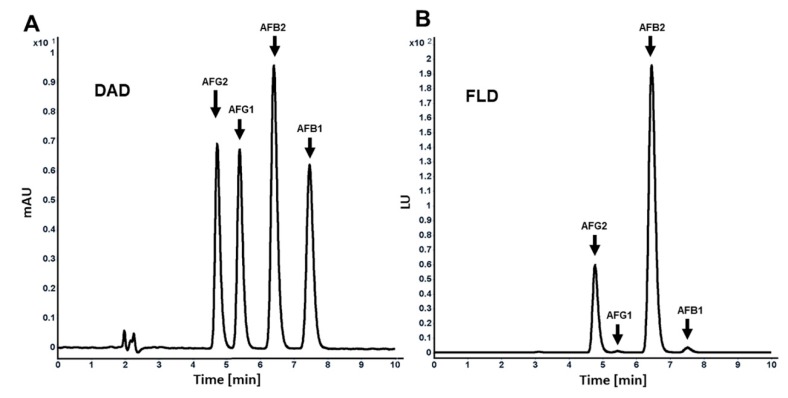
High-performance liquid chromatography (HPLC) chromatograms of the standard solution containing four aflatoxins (100 ng/mL each of AFB1, AFB2, AFG1, and AFG2) detected by diode array (DAD) (**A**) and fluorescence (FLD) (**B**).

Method validation is a crucial prerequisite to performing an analysis [23]. Several methods are available for analysis of AFs in food and feed that have been validated and accepted by official authorities, such as the European Committee for Standardization, the Association of Official Analytical Chemists (AOAC), and the International Organization for Standardization (ISO). Here, we employed a reverse-phase chromatography for the analysis of AFs by using a nonpolar bonded silica surface column and a polar mobile phase. With this reversed phase mode, AFs were eluted in the order of AFG2, AFG1, AFB2, and AFB1 (Figure 2A,B). This order was confirmed by comparing the obtained retention times in an AF mixture with the retention times of the individual AFs. All separated AFs were then detected by DAD and FLD detectors, connected in series, at parts per billion (ppb; ng/mL) concentrations (Figure 2A,B). It needs to be noted that, in using the FLD detector, AFG2 and AFB2 could be detected even at lower levels, as they fluoresce 40-fold more than AFB1 and AFG1 (Figure 2B). The LOQ is defined as the minimum concentration or mass of analyte in a given matrix that can be reported as a quantitative result with a certain level of precision [24]. On the contrary, the LOD is defined as the lowest concentration of the analyte that can be detected, but not necessarily quantitated, under the stated experimental conditions [25]. The LOD and LOQ for all AFs as detected by the UV detector was 1.0 ng/mL and 2.5–5.0 ng/mL, respectively. Using an FLD detector, the LOD and LOQ for AFB1 and AFG1 were 1.0 ng/mL and 2.5 ng/mL, respectively. Importantly, the LOD and LOQ for AFB2 and AFG2 using our method were 0.01 and 0.025 ng/mL, respectively. This method was designed for detection and quantification of aflatoxins mixture in laboratory cultures medium of growing fungi and it is not intended to use for food or feed for regulatory purpose. We found that DAD could at most detect as low as 1.0 ng/mL and quantify as low as 2.5–5.0 ng/mL for all aflatoxins. However, we injected several concentrations below 2.5 ng/mL to check the sensitivity of the FLD for detection of aflatoxins, specifically B2 and G2. We found that AFB2 and AFG2 could be easily detected, as expected and previously proved, at parts per trillion (ppt) level by FLD because of the absence of a double bond in the furan ring. To the best of our knowledge with fungal and fungal genetic studies, 1.0–5.0 ng/mL as LOD is sufficient to help researchers to distinguish between aflatoxigenic and non-aflatoxigenic strains, as well as the relative amounts of aflatoxins B and G produced between aflatoxigenic strains.

**Figure 3:**
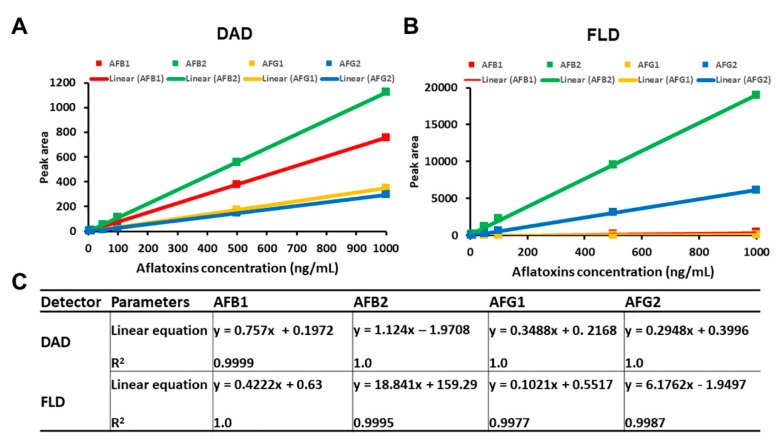
Calibration of aflatoxins. (**A**) Calibration curves of standard aflatoxin solutions (AFB1, AFB1, AFG1, and AFG2) over the concentrations of 5, 10, 50, 100, 500, and 1000 ng/mL as detected by DAD. (**B**) Those detected by FLD. (**C**) Linear relationship between aflatoxin concentrations and peak areas in the range of 5 to 1000 ng/mL. Correlation coefficient (*R^2^*) and regression equation values were determined by plotting area values (*y*-axis) against aflatoxin concentration (*x*-axis).

Selectivity is defined as the ability to separate the analyte from other components (including impurities) that may be present in the sample [26]. Our method demonstrated a good separation ability and selectivity that allowed simultaneous quantification of four different AFs in the culture medium without interference between the AFs. Both detection methods (DAD and FLD) were able to differentiate the AF peaks in the same HPLC run with minimal background interference. In order to demonstrate a proportional relationship of response versus AF concentrations over the working range, the linearity of the method was tested from the calibration curves using seven points over the range of 5.0–1000 ng/mL for each AF and defined using the correlation coefficient (coefficients of determination, *R^2^*) and the slope. Calibration curves were constructed by plotting the peak area (*y*) versus the concentrations of the AFs (*x*) (Figure 3A,B). Calibration curves fitted by linear regression showed *R^2^* ranging from 0.9987 to 1.0 for both detectors, indicating an excellent linearity for all four AFs (Figure 3C).

The fraction or percentage of the analyte that is recovered when the test sample is analyzed using the entire method is referred to as the method recovery [27]. Table 1 shows the percentage of AF recovery at a low, three-point intermediate, and high concentration levels spiked in three culture conditions. Recovery of AFs in solid, submerged, and slant culture states showed similar retention times with an overall average recovery of 76%–88%, 77%–88.4%, and 77%–86%, respectively. All spiked samples were detected by both DAD and FLD in a series manner, and the mean of both was calculated. This recovery range was within the guideline of acceptable recovery limits of AOAC and the Codex Alimentarius. The AOAC guideline for the acceptable recovery at the 10 ng/mL level is 70%−125%. The Codex Alimentarius acceptable recovery range is 70%−110% for a level of 10−100 ng/mL and 60%−120% for a level of 1−10 ng/mL. The repeatability of the method for AF analysis, as evaluated by the percentage of the RSD, ranged from 0.8% to 8.9%. These values agree with the AOAC guideline for a validated analytical method. The AOAC guidelines for acceptable repeatability (RSD) at 10 ng/mL are less than 15% and less than 8% at 1000 ng/mL.

**Figure 4:**
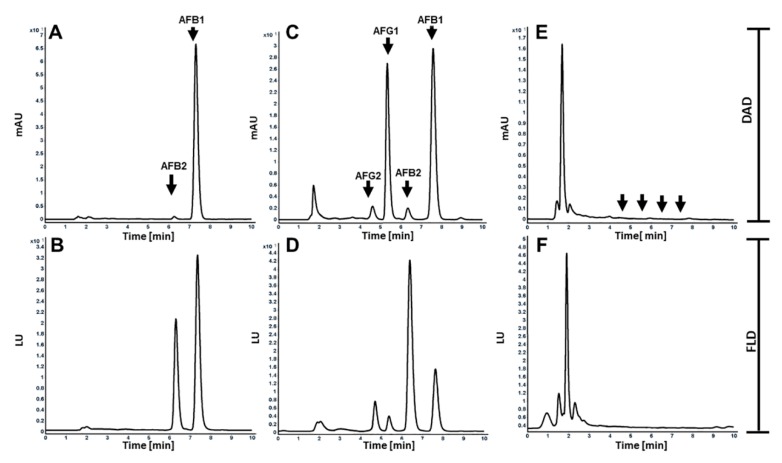
Analyses of aflatoxins from three different *Aspergillus* species. Representative HPLC chromatograms of aflatoxins in five-day potato dextrose agar (PDA) solid culture of *Aspergillus* *flavus* NRRL 3357 detected by DAD (**A**) and FLD (**B**), in five-day potato dextrose broth (PDB) submerged culture of *A. parasiticus* NRRL 2999 detected by DAD (**C**) and FLD (**D**), and in five-day PDB slant culture of *Aspergillus oryzae* M2040 detected by DAD (**E**) and FLD (**F**) are shown. Note that no aflatoxins are detectable from the culture of this food-grade strain.

To validate our method, AFs were extracted from known aflatoxigenic and non-aflatoxigenic *Aspergillus* strains grown in three different culture conditions. *A. flavus* NRRL 3357 was able to produce 879 and 7.8 ng/mL of AFB1 and AFB2 (Figure 4A,B), respectively, when the fungus was cultured in solid agar with a total amount of 13,302 ng per plate. This strain produced 2041.9 and 221.1 ng/mL of AFB1 and AFB2, respectively, when grown in liquid culture medium. On a slant cultivation, NRRL 3357 yielded 1100 and 11.49 ng/mL of AFB1 and AFB2, respectively. *A. parasiticus* NRRL 2999 was able to produce 398.27, 2.98, 207.8, and 10.19 ng/mL of AFB1, AFB2, AFG1, and AFG2, respectively, when it was inoculated onto an agar plate with a total amount of 9288.6 ng per plate. In liquid cultivation, this fungus was able to produce 508.2, 24.21, 339.3, and 42.6 ng/mL for AFB1, AFB2, AFG1, and AFG2 (Figure 4C,D), respectively. It was able to produce 310, 10.1, 437.86, and 37.66 ng/mL of AFB1, AFB2, AFG1, and AFG2, respectively, when the fungus was cultured in a slant tube. No peaks were detected within the expected retention times for *Aspergillus oryzae* NRRL 3483 grown on any of three cultivation mediums. Representative chromatograms of *A. oryzae* NRRL 3483 grown in slant cultivation medium are shown in Figure 4E,F. In addition, *A. oryzae* M2040 and *A. oryzae* NRRL RIB40 were unable to produce any types of AFs when grown in different culture medium, as shown in previous studies [28,29].

In summary, in this work, we report a HPLC method coupled with DAD and FLD detectors that would be the first and only validated tool for simultaneous quantitation of AFB1, AFB2, AFG1, and AFG2 in three different laboratory culture conditions. This was an effective tool for quantitative screening of AFs in diverse *Aspergillus* strains. Chloroform was used as the extraction solvent to avoid emulsion formation—the mixture separates into two layers with AFs in the chloroform layer, thus reducing toxin loss and leaving other compounds in the aqueous layer. The extraction and cleanup procedures can be performed in less than 10 min and do not require the use of large amount of solvent or immune-affinity columns (IAC). The HPLC analysis is to be performed without any pre- or post-column derivatization reagents or any fluorescent enhancers. Peaks of the four AFs are separated in less than 10 min with high selectivity, linearity, and recovery. Finally, our method provides sufficient sensitivity to enable AF detection within mixtures at ppb levels for AFB1 and AFG1, and at parts per trillion (ppt) levels for AFB2 and AFG2 via FLD detection. In addition, our method can by readily available and easily applied in most mycology laboratories.

## 3. Materials and Methods

### 3.1. Chemicals and Materials

Individual AF standards of AFB1, AFB2, AFG1, and AFG2 were purchased from Sigma Chemical Co. (St. Louis, MO, USA). HPLC-grade acetonitrile and methanol (Merck, Darmstadt, Germany) were used for the preparation of the mobile phase. Analytical grade chloroform (Fischer Scientific, Leicestershire, United Kingdom) was used for extraction of aflatoxins. Reverse osmosis (RO) water was used for the preparation of the mobile phase, culture medium, and 0.1% Tween solution. Membrane filters (0.45 μm with 47 mm diameter), polytetrafluoroethylene (PTFE) syringe filters (0.45 μm with 17 mm diameter) and Polysorbate 80 (Tween-80) were purchased from Thermo Fisher Scientific (Rockwood, TN, USA). Both potato dextrose agar (PDA) and potato dextrose broth (PDB) were purchased from BD Difco Laboratories (Sparks, MD, USA).

### 3.2. Preparation of Aflatoxin Standards

Standard solutions of each of the four representative AFs AFB1, AFB2, AFG1, and AFG2 (Sigma) were prepared in acetonitrile at a final concentration of 10 μg/mL (part per million; ppm) according to the Association of Official Analytical Chemists (AOAC) method [30]. To prepare 10 μg/mL individual AF stock standard solutions, 10 mg of each AF was weighed into a separate 100 mL volumetric flask. Acetonitrile (50 mL) was added to each flask, mixed, and further added to the mark and mixed again. Then, 10 mL of this solution was transferred into another 100 mL volumetric flask and diluted to the mark with acetonitrile. Working solutions (individual or mixture) were prepared in acetonitrile and stored at −20 °C in amber glass vials for the study period (up to 3 months). The AF standard solutions used for the HPLC calibration curve were prepared by further dilution of the working solutions with the mobile phase.

### 3.3. Fungal Strains

*Aspergillus flavus* NRRL 21,882 (Afla-Guard), in which the entire AF biosynthetic gene cluster was deleted, was used as a non-aflatoxigenic strain [28,31] for the recovery experiments. The food-grade *Aspergillus oryzae* RIB40 [29], *A. oryzae* M2040 [28], and *A. oryzae* NRRL 3483 were used as non-aflatoxigenic strains. The aflatoxigenic strain *A. flavus* NRRL 3357 was used as a positive control, as it is a well-known AFB1 and AFB2 producer in lab and fields [3]. In addition, *A. parasiticus* NRRL 2999 was used as a positive control for AFB and AFG production [32]. All fungal strains were maintained on potato dextrose agar (PDA) medium (containing 4 g potato starch, 20 g glucose, and 15 g agar in 1 L of distilled water) at 4 °C. This medium, which has a high carbohydrate content and an acidic pH (5.1), was selected because it enhanced mold growth and aflatoxin production [28]. To prepare inoculum for these fungi, all were grown on PDA for 7 days at 30 ± 2 °C. *A. parasiticus* NRRL 2999 was grown on PDA for 7 d at 25 ± 2b °C. Spores were harvested from individual cultures using 0.1% Tween-80 solution. Asexual spores (conidia) were counted with a hemocytometer, and numbers were adjusted to 1 × 10^8^ conidia/mL with sterile RO water. Fungal spore suspensions were stored at 4 °C and used within one week of preparation.

### 3.4. Culture Conditions

Fungal spore suspensions were used as a source of the inoculum for all cultivation states. *A. flavus* NRRL 21,882 was inoculated into three cultivation states: submerged cultivation, solid state, and semi-solid (slant). The incubation temperature for all strains except *A. parasiticus* (25 ± 2 °C) was 30 ± 2 °C. For submerged cultivation, 250 mL Erlenmeyer flasks were filled with 100 mL PDB and inoculated with fungal strains at a 5 × 10^5^ conidia/flask. All flasks were incubated at 30 ± 2 °C with shaking at 220 rpm for 5 days. For solid state cultivation, Petri dishes (100 × 15 mm) containing about 25 mL of PDA were inoculated with fungal strains (5 × 10^5^ conidia/plate) using a micropipette, and the spores were spread onto the plate surface by streaking. The plates were inverted and incubated at 30 ± 2 °C for 5 days. For slant cultivation, screwcap 25 mL glass test tubes were filled with 10 mL of PDB and inoculated with fungal strains (5 × 10^5^ conidia/tube) using a micropipette, and then the spores were streaked back and forth from the bottom to the top of the slant using an inoculating loop. The tubes were placed in a rack and positioned at a 45° angle in the incubator at 30 ± 2 °C for 5 days.

### 3.5. Extraction of Aflatoxins from Cultures

**Figure 5:**
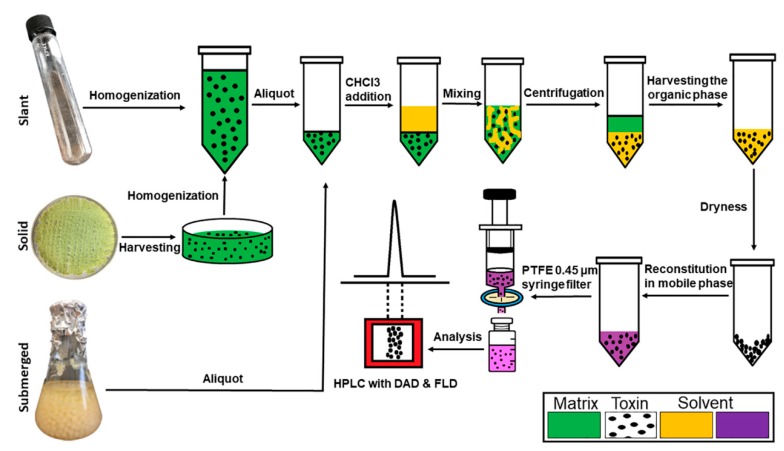
A process flow diagram for the extraction of aflatoxins from three different culture conditions.

A flow diagram for the extraction of aflatoxins from three different culture conditions is shown in Figure 5. Aflatoxins (B1, B2, G1, and G2) were extracted from the submerged culture by liquid–liquid extraction. Briefly, 1.0 mL aliquot of the fungal culture broth was mixed with 1.5 mL of chloroform in a 15 mL centrifuge tube and vigorously shaken by hand for 10 s followed by vortexing for 30 s. The mixture was then centrifuged for 2.5 m at 5000× *g*. The organic phase in the lower layer was transferred to a new glass vial. The sample residue was re-extracted with another 1.5 mL of chloroform to recover traces of AFs, which might have been present after the first extraction. The two chloroform extracts were combined and evaporated to complete dryness under a gentle stream of air. The dried extract was reconstituted with 1.0 mL of mobile phase. AFs from solid culture were extracted by adding 15.0 mL of 0.1% Tween-80 solution; the conidia were then harvested by gently scraping the top layer and collected into a 50 mL centrifuge tube. Spore suspension was homogenized by vortexing for 30 s. One mL of this suspension was transferred to a new centrifuge tubes (15 mL), and 1.5 mL of chloroform was added. The extractions were performed as described above for the liquid culture. For the liquid slant culture, the cultivated tubes were vortexed for 30 s, and 1.0 mL of the suspension was transferred to a new centrifuge tube (15 mL). Then, 1.5 mL of chloroform was added to the tubes and treated as described above. All samples were filtered into HPLC vials through a PTFE 0.45 μm syringe filter prior to HPLC analysis.

### 3.6. HPLC Analysis of Aflatoxins

Samples were analyzed for AFB1, AFB2, AFG1, and AFG2 using a model 1100 HPLC system consisting of a degasser, an autosampler, and a quaternary pump, and equipped with a diode array 1260 Infinity (DAD) and fluorescence 1260 Infinity II (FLD) detectors connected in series (Agilent Technologies, Santa Clara, CA, USA). The separation was performed via a Zorbax Eclipse XDB-C18 4.6 × 150 mm, 3.5 μm column with a temperature set at 30 °C. Samples were monitored at a wavelength of 365 nm for UV detection and at 365 nm excitation and 450 nm emission for FLD detection. The samples were eluted with the mobile phase of water (H_2_O)/methanol (CH_3_OH)/acetonitrile (CH_3_CN) (50:40:10) at a flow rate of 0.8 mL/m. The mobile phase was degassed and filtered through a membrane filter (47 mm × 0.45 μm) prior to use. The injection volume was 100 μL. Peak areas of AFs were recorded and integrated using the ChemStation software (Agilent).

### 3.7. Method Validation

The method employed for the extraction and simultaneous analysis of AFB1, AFB2, AFG1, and AFG2 in the laboratory culture conditions was validated according to the AOAC Guidelines Appendix F [33], with slight modifications, by determining the recovery, precision, selectivity, linearity, and the sensitivity. A mixture of known concentrations of four AFs (500, 100, 50, 10, and 2.5 of each) were spiked into blank culture samples (submerged, slant, and solid state) for the recovery validation. Each concentration was spiked in triplicate, and the experiments were repeated three times within a day and repeated in 3 consecutive days by the same operator. Precision was demonstrated as repeatability, which was evaluated by calculating the relative standard deviation (% RSD) of the spiked toxins repeated within 1 day and over 3 consecutive days. Blank samples were prepared by inoculating non-aflatoxin-producing *A. flavus* NRRL 21,881 in submerged, solid, and slant cultures. The samples were then harvested, and AFs were extracted and analyzed by HPLC coupled with DAD and FLD. The selectivity of this method was assured as there were no interfering chromatographic peaks corresponding to the retention time of the four AFs. The linearity was demonstrated for the AFs in the range of 2.5 to 1000 ppb in three replicates. The calibration standard of each concentration was constructed using the peak-area ratio of the AFs versus the concentration of the analytes. The linear relationship was evaluated by the correlation coefficient, *y*-intercept, and slope of the regression line. The sensitivity of the method was determined by measuring the LOD and the LOQ on the basis of a signal-to-noise ratio (S/N) of 3:1 and 6:1, respectively.

### 3.8. Aflatoxin Analyses from Cultures of Aspergillus Species

To verify the protocol of the AF quantification from the cultures, two common strains of aflatoxin-producing fungi, *A. flavus* NRRL 3357 and *A. parasiticus* NRRL 2999, were used and tested for aflatoxin production. In addition, three of the non-aflatoxin producing *Aspergillus* strains were used as a negative control: *A. oryzae* M2040, *A. oryzae* NRRL RIB40, and *A. oryzae* NRRL 3483. Fungal strains were grown in three culture conditions in triplicate, as mentioned previously; samples were then harvested after 5 days of incubation, and AFs were extracted and analyzed by HPLC coupled with DAD and FLD.

### 3.9. Statistical Analyses

The method was optimized and validated with a statistical treatment to increase the AF recovery and to save time and reagent waste. AFs peaks were simultaneously separated with no interfering. Significance (*p* < 0.05) of the data was analyzed by using a Student’s *t*-test. The relative standard deviation (RSD) was calculated using Equation (1).

RSD = Si × 100/x(1)

S = standard deviation, and x = mean of the data.

## Figures and Tables

**Table 1:** Recovery (%) of spiked aflatoxins from three culture methods (solid, submerged, and slant cultures); mean with (RSD) in percentages.

Spiked Levels (ng/mL)	Recovery of Aflatoxins (%)			
	AFB1	AFB2	AFG1	AFG2
	Solid Culture			
500	86.1 (3.6)	88.2 (4.2)	85.6 (5.4)	86.4 (5.4)
100	79.9 (1.2)	87.0 (0.8)	81.7 (4.1)	85.2 (1.9)
50	79.7 (1.5)	79.6 (3.4)	79.8 (5.9)	82.8 (5.2)
10	78.1 (3.2)	81.6 (6.4)	78.8 (7.6)	79.0 (1.5)
2.5	79.9 (1.2)	78.1 (5.2)	77.2 (7.7)	76.1 (4.6)
	Submerged Culture			
500	87.5 (3.1)	88.4 (4.2)	84.4 (4.7)	86.4 (5.0)
100	81.4 (3.8)	83.3 (4.4)	78.6 (5.1)	81.1 (4.1)
50	82.2 (3.2)	81.2 (4.1)	80.7 (2.3)	78.2 (4.2)
10	83.2 (6.1)	83.4 (5.4)	84.1 (6.7)	82.4 (4.9)
2.5	77.6 (2.3)	83.4 (3.4)	78.2 (1.7)	78.3 (1.5)
	Slant Culture			
500	85.4 (1.4)	85.2 (3.4)	86.3 (2.6)	83.4 (0.9)
100	85.2 (3.6)	84.3 (4.2)	81.7 (3.8)	81.2 (1.3)
50	85.7 (8.9)	78.2 (3.2)	81.1 (2.7)	79.4 (1.1)
10	84.2 (7.1)	83.2 (5.7)	82.2 (5.0)	83.4 (7.3)
2.5	79.2 (3.1)	82.2 (3.1)	78.5 (0.9)	77.3 (7.2)

## References

[1] Ostry V., Malir F., Toman J., Grosse Y. (2017). Mycotoxins as human carcinogens-the IARC Monographs classification. Mycotoxin Res..

[2] Loomis D., Guha N., Hall A.L., Straif K. (2018). Identifying occupational carcinogens: an update from the IARC Monographs. Occup. Environ. Med..

[3] Klich M.A. (2007). Aspergillus flavus: the major producer of aflatoxin. Mol. Plant Pathol..

[4] Alshannaq A., Yu J.-H. (2017). Occurrence, Toxicity, and Analysis of Major Mycotoxins in Food. Int. J. Env. Res. Public Health.

[5] Wu F., Guclu H. (2012). Aflatoxin regulations in a network of global maize trade. Plos One.

[6] Weaver M.A., Mack B.M., Gilbert M.K. (2019). Genome Sequences of 20 Georeferenced Aspergillus flavus Isolates. Microbiol. Resour. Announc..

[7] Van der Fels-Klerx H.J., Vermeulen L.C., Gavai A.K., Liu C. (2019). Climate change impacts on aflatoxin B1 in maize and aflatoxin M1 in milk: A case study of maize grown in Eastern Europe and imported to the Netherlands. Plos One.

[8] Medina A., Rodriguez A., Magan N. (2014). Effect of climate change on Aspergillus flavus and aflatoxin B1 production. Front. Microbiol..

[9] Assuncao R., Martins C., Viegas S., Viegas C., Jakobsen L.S., Pires S., Alvito P. (2018). Climate change and the health impact of aflatoxins exposure in Portugal—An overview. Food Addit. Contam. Part A.

[10] Hruska Z., Yao H., Kincaid R., Brown R., Cleveland T., Bhatnagar D. (2014). Fluorescence Excitation–Emission Features of Aflatoxin and Related Secondary Metabolites and Their Application for Rapid Detection of Mycotoxins. Food Bioprocess Technol..

[11] Fente C.A., Ordaz J.J., Vázquez B.I., Franco C.M., Cepeda A. (2001). New additive for culture media for rapid identification of aflatoxin-producing Aspergillus strains. Appl. Env. Microbiol..

[12] Maragos C.M., Appell M., Lippolis V., Visconti A., Catucci L., Pascale M. (2008). Use of cyclodextrins as modifiers of fluorescence in the detection of mycotoxins. Food Addit. Contam. Part A.

[13] Sharma A., Khan R., Catanante G., Sherazi A.T., Bhand S., Hayat A., Marty L.J. (2018). Designed Strategies for Fluorescence-Based Biosensors for the Detection of Mycotoxins. Toxins.

[14] Drott M.T., Fessler L.M., Milgroom M.G. (2019). Population Subdivision and the Frequency of Aflatoxigenic Isolates in Aspergillus flavus in the United States. Phytopathology.

[15] Camiletti B.X., Torrico A.K., Maurino M.F., Cristos D., Magnoli C., Lucini E.I., Pecci M.D.L.P.G. (2017). Fungal screening and aflatoxin production by Aspergillus section Flavi isolated from pre-harvest maize ears grown in two Argentine regions. Crop Prot..

[16] Baranyi N., Despot D.J., Palagyi A., Kiss N., Kocsube S., Szekeres A., Kecskemeti A., Bencsik O., Vagvolgyi C., Klaric M.S. (2015). Identification of Aspergillus species in Central Europe able to produce G-type aflatoxins. Acta Biol. Hung..

[17] Okoth S., De Boevre M., Vidal A., Diana Di Mavungu J., Landschoot S., Kyallo M., Njuguna J., Harvey J., De Saeger S. (2018). Genetic and Toxigenic Variability within Aspergillus flavus Population Isolated from Maize in Two Diverse Environments in Kenya. Front. Microbiol..

[18] Saldan N.C., Almeida R.T.R., Avincola A., Porto C., Galuch M.B., Magon T.F.S., Pilau E.J., Svidzinski T.I.E., Oliveira C.C. (2018). Development of an analytical method for identification of Aspergillus flavus based on chemical markers using HPLC-MS. Food Chem..

[19] Abbas H.K., Zablotowicz R.M., Weaver M.A., Horn B.W., Xie W., Shier W.T. (2004). Comparison of cultural and analytical methods for determination of aflatoxin production by Mississippi Delta Aspergillus isolates. Can. J. Microbiol..

[20] Sadhasivam S., Britzi M., Zakin V., Kostyukovsky M., Trostanetsky A., Quinn E., Sionov E. (2017). Rapid Detection and Identification of Mycotoxigenic Fungi and Mycotoxins in Stored Wheat Grain. Toxins.

[21] Gell R.M., Carbone I. (2019). HPLC quantitation of aflatoxin B1 from fungal mycelium culture. J. Microbiol. Methods.

[22] Garcia M., Blanco J.L., Suarez G. (1994). Aflatoxins B1 and G1 solubility in standard solutions and stability during cold storage. Mycotoxin Res..

[23] Rogers H.A. (2013). How Composition Methods Are Developed and Validated. J. Agric. Food Chem..

[24] Şengül Ü. (2016). Comparing determination methods of detection and quantification limits for aflatoxin analysis in hazelnut. J. Food Drug Anal..

[25] Currie L.A. (1999). Detection and quantification limits: origins and historical overview1Adapted from the Proceedings of the 1996 Joint Statistical Meetings (American Statistical Association, 1997). Original title: “Foundations and future of detection and quantification limits”. Contribution of the National Institute of Standards and Technology; not subject to copyright.1. Anal. Chim. Acta.

[26] Danzer K. (2001). Selectivity and specificity in analytical chemistry. General considerations and attempt of a definition and quantification. Fresenius’ J. Anal. Chem..

[27] Trucksess M.W., Weaver C.M., Oles C.J., Fry F.S., Noonan G.O., Betz J.M., Rader J.I. (2008). Determination of aflatoxins B1, B2, G1, and G2 and ochratoxin A in ginseng and ginger by multitoxin immunoaffinity column cleanup and liquid chromatographic quantitation: collaborative study. J. Aoac Int..

[28] Alshannaq A.F., Gibbons J.G., Lee M.K., Han K.H., Hong S.B., Yu J.H. (2018). Controlling aflatoxin contamination and propagation of Aspergillus flavus by a soy-fermenting Aspergillus oryzae strain. Sci. Rep..

[29] Rank C., Klejnstrup M.L., Petersen L.M., Kildgaard S., Frisvad J.C., Held Gotfredsen C., Ostenfeld Larsen T. (2012). Comparative Chemistry of Aspergillus oryzae (RIB40) and A. flavus (NRRL 3357). Metabolites.

[30] AOAC Official Methods of Analysis (2005). Preparation of Standards for Aflatoxins.

[31] Dorner J.W., Lamb M.C. (2006). Development and commercial use of afla-Guard((R)), an aflatoxin biocontrol agent. Mycotoxin Res..

[32] Wilson D.M., King J.K. (1995). Production of aflatoxins B1, B2, G1, and G2 in pure and mixed cultures of Aspergillus parasiticus and Aspergillus flavus. Food Addit. Contam..

[33] AOAC Official Methods of Analysis (2016). Guidelines for Standard Method Performance Requirements.

